# Sleep Phenotypes of Caregivers for Persons Living With Dementia

**DOI:** 10.1093/geroni/igab046.1463

**Published:** 2021-12-17

**Authors:** Glenna Brewster, Christina Pierpaoli, Fayron Epps, Kalisha Bonds Johnson, Kate Yeager

**Affiliations:** 1 Emory University, Stonecrest, Georgia, United States; 2 University of Alabama, Tuscaloosa, Alabama, United States; 3 Emory University, Atlanta, Georgia, United States

## Abstract

Sleep disturbance is prevalent among caregivers of people living with dementia. Gaps exist regarding when caregivers begin to experience sleep disturbance along their caregiving trajectory. This study aimed to identify and describe phenotypes of current caregivers’ sleep patterns before and during caregiving and describe caregivers’ perception of their current sleep quality relative to their pre-caregiving sleep. We conducted semi-structured interviews with 19 caregivers participating in a larger, randomized controlled trial. Interviews were about caregivers’ sleep patterns and were conducted after caregivers completed the first 6 months of the study. Interviews were audio-recorded using a videoconferencing platform and ranged from 20 to 45 minutes. We conducted thematic analysis of the interview transcripts. Four distinct caregiver-sleep phenotypes emerged from the qualitative data: Changed and Dissatisfied, Changed and Satisfied, Unchanged and Dissatisfied, and Unchanged and Satisfied. Caregivers whose sleep was categorized as Changed experienced a difference in their pre-caregiving sleep, usually from good to poor sleep. Caregivers whose sleep was Unchanged had poor sleep pre-caregiving and continued to have poor sleep during caregiving. Caregivers also reported being Satisfied or Dissatisfied with their current sleep pattern, defined in terms of daily distress and impairment. These 4 subtypes highlight the heterogeneity of caregivers’ sleep experiences and debut a useful clinical framework with which to identify, categorize, and target caregivers at risk for sleep disturbance. Identifying caregivers’ sleep phenotypes will enable healthcare providers to determine caregivers’ needs and readiness for interventions.

